# Evaluation of hair growth promoting activity of *Phyllanthus niruri*

**Published:** 2015

**Authors:** Satish Patel, Vikas Sharma, Nagendra S. Chauhan, Mayank Thakur, Vinod Kumar Dixit

**Affiliations:** 1 *Department of Pharmaceutical Sciences, Dr. Hari Singh Gour Vishwavidyalaya Sagar- 470003 (M.P.),India*; 2 *Institute for Laboratory Medicine Clinical Chemistry and Pathobiochemistry, Charite Universitäts Medizin, Campus Virchow Klinikum, Augustenburgerplatz 1, Berlin, Germany*

**Keywords:** *5α-reductase*, *Alopecia*, *Finasteride*, *Hair growth*, *Phyllanthus niruri*

## Abstract

**Objective:**

This study was designed to investigate the potential *Phyllanthus niruri *(*P. niruri ) *extracts in promotion of hair growth.

**Materials and Methods:**

Here, we studied the hair growth promoting activity of petroleum ether extract of *P. niruri *following its topical administration. Alopecia was induced in albino rats by subcutaneous administration of testosterone for 21 days. Evaluation of hair loss inhibition was done by concurrent administration of extract and monitoring parameters like follicular density, anagen/telogen (A/T) ratio and histological observation of animal skin sections. Finasteride solution was applied topically as standard. *In vitro* experiments were also performed to study the effect of extract on the activity of 5α-reductase enzyme

**Results:**

Groups treated with petroleum ether extract of plant showed hair re-growth as reflected by follicular density, A/T ratio and skin sections. Histopathology and morphologic observations of hair re-growth at shaved sites showed active follicular proliferation. *In vitro* experiments results showed inhibitory activity of petroleum ether extract on type-2 5α-reductase enzyme and an increase in the amount of testosterone with increasing concentrations.

**Conclusion:**

It could be concluded that petroleum ether extracts of *P. niruri *might be useful in the treatment of testosterone-induced alopecia in the experimental animal by inhibiting 5α-reductase enzyme.

## Introduction

Alopecia is a dermatological disorder that has been known for more than thousand years. It is seen all over the world and affects approximately 0.2-2% of the world population (Sperling, 2001 ▶). Androgenetic alopecia (AGA) is an androgen-linked condition in genetically prone individuals, which exerts influence on 50% of the male population (Otberg et al. 2007 ▶). A number of genetic and environmental factors play a role in causing AGA. AGA is a frequent form of alopecia in which androgens progressively convert normal-sized scalp hair follicles to miniaturized hair follicle (Olsen, 1994 ▶). Dermal papilla cells are mainly affected by 5α-dihydrotestosterone (5α-DHT) among all androgens. It is produced from testosterone in dermal papilla cells by catalytic action of 5α-reductase type-2 enzyme (Nutbrown et al. 1995 ▶). The 5α-reductase type-2 enzyme plays a vital role through intrafollicular conversion of testosterone to 5α-DHT, and hair loss is characterized by shortening of the anagen phase and miniaturization of hair follicles resulting in thinner and shorter hairs (Pérez-Ordeals, 2005 ▶).

 The pathogenesis entails androgen-mediated miniaturization of terminal hairs into vellus hairs in affected regions of the scalp. A number of medical treatments and therapies that succeeded to hair loss have become available in recent years, and surgical treatments are constantly being developed. Presently, minoxidil and finasteride are two synthetic drugs used in the treatment of androgenic alopecia (Goodman and Gilman, 1996 ▶; Headington and Novak, 1984 ▶). Natural products have been widely used in hair care industry and the search for natural remedies is being interminably promoted.

*Phyllanthus niruri* is a small plant, which grows up mainly in tropical and subtropical regions in Central and South American countries, India and East Asia. It is one of the most important medicinal plants used by people in these countries for treatment of jaundice, asthma, hepatitis, urolithic disease, fever, malaria, stomachache and tuberculosis (Unander et al. 1991 ▶). Chemical characterization of this plant has been carried outand several constituents were isolated such as lignans, alkaloids, flavonoids, tannins, phthalic acid, gallic acid, and terpenoids (Balawantei al. 1986 ▶; Calixto et al. 1998 ▶). Therefore, the present study was designed to assess the *in vitro* and *in vivo *effect of *P. niruri *extracts on testosterone-induced hair loss and to elucidate its possible mechanism of action guesstimating its inhibitory effect on 5α-reductase type-2 enzyme.

## Materials and Methods

**Collection and authentication**

The whole plants of *P. niruri* were collected in the period of September-October 2010 from forests surrounding our university campus, Sagar (M.P.), India and authenticated by Dr. P.K Tiwari, Department of Botany, Dr. H.S.Gour University Sagar (M.P.), India (Herbarium no. Bot/Her/1329). Plants were dried out under sunlight and reduced to a coarse powder. The extract of the powdered aerial part of *P. niruri *was prepared using petroleum ether and soxhlet extraction method.

***In vivo*** **studies on hair growth animals**

Eighteen male Swiss albino rats (6-8 months age, 130-140g) with no earlier drug treatment were used. The animals are maintained under conventional conditions with food and water provided *ad libitum*. Care and handling of the animals were in accordance with the guidelines of CPCSEA, India. The Institutional Ethical Committee of Dr H.S. Gour University (Reg. No. 319/01/ab/CPCSEA) approved the protocol for all animal experiments.

**Solutions**

Testosterone solution (1%) was prepared in arachis oil. Ethanol: Propylene glycol: water (8:1:1) was used asthe vehiclein which the extracts (2%) and the standard (Finasteride) solution (2%) were prepared. 

**Grouping of animals and treatment**

The animals were randomly divided into three groups of six male Swiss albino rats and were treated as follows:

 Group I: Testosterone solution (subcutaneous,S.C.) + Vehicle (Topically)

 Group II: Testosterone solution (S.C.) + finasteride solution (2%) (Topically)

 Group III: Testosterone solution (S.C.) + petroleum ether extract (2%) of *P. niruri* (Topically)

 The Matia’s method was followed with insignificant modifications (Matias et al. 1989 ▶; Pandit et al. 2008 ▶). Daily 0.1 ml of testosterone(S.C.)was administered to rats in all groups. Animals of groups I, II, III were given topical application of vehicle, finasteride and petroleum ether extract of *P. niruri* respectively.

 Daily 0.2 ml(approximate volume)of the solutions or vehicle was administered topically on back skin for 20 days. Hair growth activity of extract was perceived by observing difference in hair growth in each group by visual observation. In skin biopsy cyclic phase of hair follicles anagen and telogen and hair follicles number were determined and the A/T ratio (anagen/telogen) was calculated using ocular micrometer. Rats from each group were selected erratically and sacrificed on day 21. In skin biopsy, the balding site of each group of rats, and samples of skin were kept in phosphate-buffered formalin for paraffin sectioning. Vertical sections (3–4μm) were cut parallel to the direction of hair growth and stained with haematoxylin and eosin.

**Enzymatic activity**

 Adult male goat prostate (7.5 g) was homogenate with sodium phosphate buffer solution at pH 6.5. Supernatant containing enzyme was collected after centrifugation of homogenate (Pandit et al. 2008 ▶). Fresh suspension was used during the reaction. Testosterone (10mg/ml), petroleum ether extracts (8.5mg/ml) and finasteride (10mg/ml) solution was prepared in ethanol (95%) with gentle heating if required. EDTA solution (10 mg/ml) was prepared in distilled water.

Bradford method was used to reveal the concentrations of enzyme in the suspension (Bradford, 1976 ▶). Optimum concentration of enzyme was determined by keeping the concentration of substrate constant and changing the concentration of enzyme. Optimum concentration of enzyme is the point at which it attained the highest velocity and the highest level of free enzyme able to interact with substrate or at which enzyme show maximum activity.

Reaction mixture (1ml) comprises of sodium phosphate buffer (pH 6.5), testosterone solution (1mg or 0.1ml) and enzyme solution (0.1-0.9 ml). Then, the reaction mixture was incubated at 37 ºC for one hour and the reaction was terminated by adding 2ml ethyl acetate. After vigorously shaking, ethyl acetate layer was separated and evaporated to dryness. The residue was dissolved in 2 ml methanol and methanolic solution was used for estimation of testosterone by high performance liquid chromatography (HPLC; Shimadzu, Column C18). 

**Inhibitory concentrations of extracts**

Inhibitory concentration of extracts was optimized using HPLC by determination of the residual testosterone content and comparison with finasteride. The column was eluted isocratically with a mobile phase of methanol:water (80: 20) at a flow rate of 1.0 ml/min (Purdon and Lehman-McKeema, 1997 ▶). For this, reaction mixture (1.5 ml) comprises of testosterone solution (0.1 mL), EDTA solution (0.1 ml), and extract/finasteride solutions (0.1–0.5 ml) for different groups, optimum amount of enzyme solution (0.6 ml) and sodium phosphate. Reaction mixture was incubated and reaction was ended by adding 3 ml of ethyl acetate. The mixture was vortexed and ethyl acetate layer was separated and evaporated to dryness. The residue was dissolved in methanol and the residual testosterone content was determined by HPLC. The IC50 values were calculated by regression equation.

## Results

***In vivo*** **studies**

**Morphologic observation**

**Figure1:**
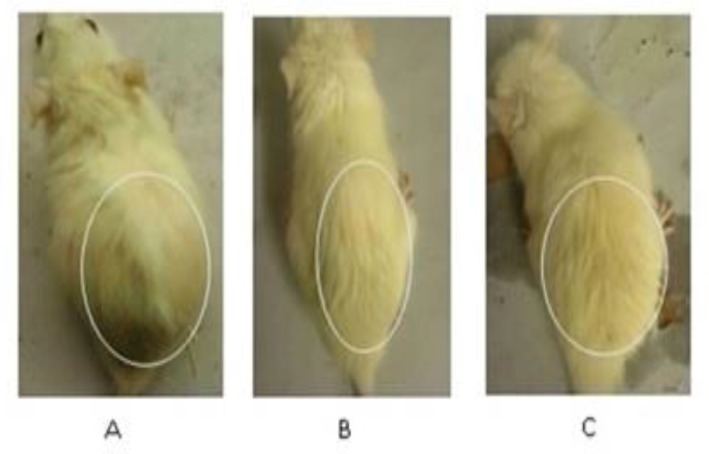
Comparison of hair loss pattern in each group. (A) Animal treated with testosterone and vehicle showing diffused alopecia. (B) Animal treated with testosterone and finasteride showed less hair loss. (C) Animal treated with testosterone and petroleum ether extract showed less hair loss

Group I rats showed alopecic symptoms; they started losing hair from day 3 from upper dorsum while animals in group II and group III did not show any symptoms on day 3. Group I (Control) rats frequently lost hair and the region of alopecia also involved the posterior back of animal. Animals in group III started losing hair from day 14 from posterior back. On day 21, at the end of study period, diffused alopecia was observed in group I while in other groups hair loss continued from posterior back and not from upper dorsum. The alopecic condition was not noticeable in these groups of animals, showing that the extract and finasteride prevented the action of testosterone and inhibited testosterone-induced hair loss (Figure 1C, Figure 1B).

**Histological observation**

**Figure 2:**
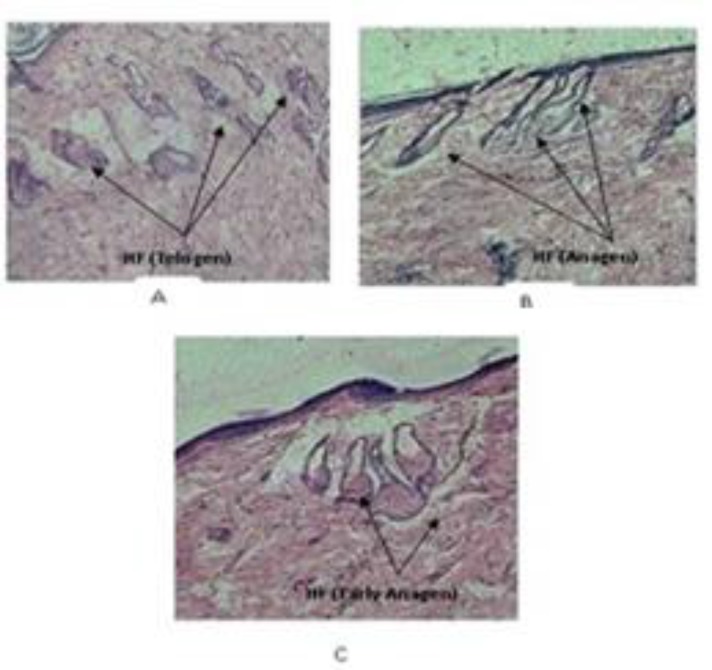
Comparison of hair loss pattern in rat skin section in each group. (A) Skin of animal treated with testosterone and vehicle. (B) Skin of animal treated with testosterone and finasteride solution. (C) Skin of animal treated with testosterone and petroleum ether extract solution

Microscopic evaluation of skin sections of group I animals revealed that due to testosterone, hair follicles miniaturized. Numerous hair follicles of group I rats were in the telogen phase (Figure 2A) as they showed characteristics of telogen follicles i.e. being short and hollow, presence of necrosis, more destroyed follicles, follicle shrinkage means diameter decreases and not deeper. In group III animals, the number of follicles in anagen phase was considerably increased and the number of follicles in telogen phase was decreased because petroleum ether extract inhibited the action of testosterone on hair follicles. Petroleum ether extract also hindered the miniaturization process. The increase in the number of hair follicles was also noted. Follicles from group II and group III rats showed characteristics of anagen follicles i.e. longer follicle hair and follicles were denser (the number increased as compared to group I), less cell necrosis, and present deeper (Figure 2B, C). Again, the number of follicles in hair growth phase increased with duration of treatment. The hair follicle density and anagen/telogen ratio (A/T ratio) was calculated (Table 1). Among the treated group, group III rats showed comparable hair growth to rats treated with finasteride. The prevalence of anagenic hair follicles specifies the reversal of androgen-induced hair loss in extract and finasteride-treated animal group.

**Table 1:** Hair follicle density and A/T ratio in sections of skin of different groups of animals

**S.No.**	**Group no.**	**Treatment**	**Hair follicular density (no./mm) **	**Anagen to Telogen**
1.	I	Testosterone (s.c)+ vehicle (topical)	1.5 ± 0.90	1 : 3.16
2.	II	Testosterone (s.c)+ 2% Finasteride solution (topical)	3.3 ± 0.77*	1.22 : 1
3.	III	Testosterone (s.c)+ 2% Pet Ether Extract of *P. niruri* solution (topical)	2.75 ± 0.75**	1.12 : 1

Value are mean ± SD, n=12

* p < 0.001,

** p < 0.005, significance versus control

*In-vivo *studies showed that there was insignificant change in prostate weights, which suggest that doses given by topical administration were not adequate to inhibit 5-reductase in prostate and inhibition was only achieved in the skin (Table 2).

***In-vitro*** **studies on enzymatic activity**

**Figure 3:**
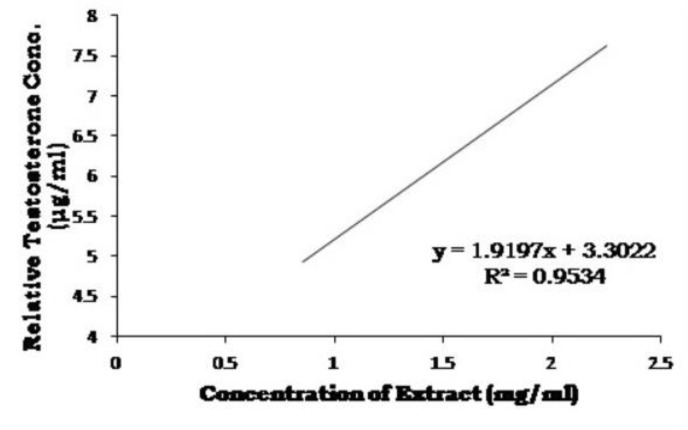
Inhibitory concentrations of petroleum ether extract of *P. niruri*

**Figure 4:**
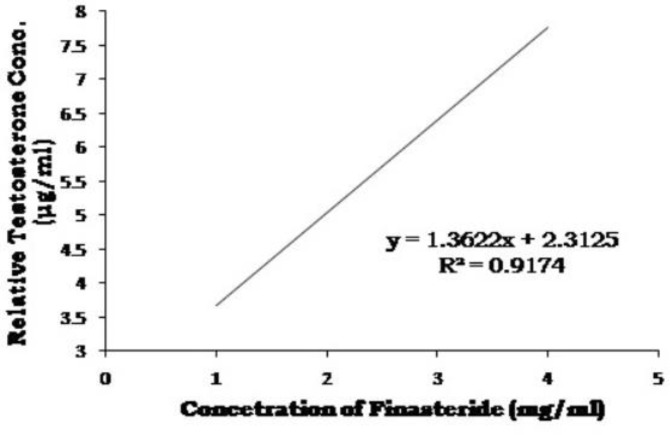
Inhibitory concentrations of finasteride

**Figure 5:**
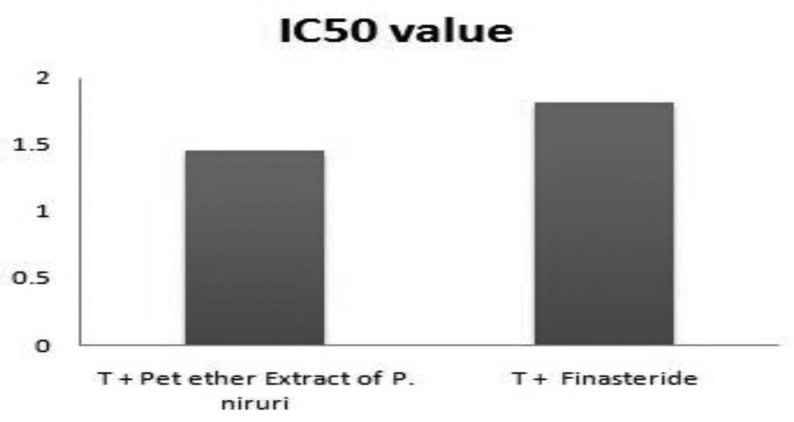
IC50 valuesforextract and finasteride

By Bradford method, the optimum amount of enzyme solution required for optimum activity was found to be 0.60 ml (213.72 g enzyme fraction). Varying concentrations of test substances were incubated with a constant amount of testosterone and enzyme in reaction mixture, and the residual testosterone content was determined after reaction with ethyl acetate. It was observed that the residual testosterone content in reaction mixture increased with increasing concentrations of petroleum ether extract of *P. niruri* and finasteride (Table 3, Table 4, Figure 3 and Figure 4). The IC50 values for extract and finasteride were obtained from inhibition curves and confirmed to be 1.46 mg/ml for petroleum ether extract and 1.81 mg/ml for finasteride, providing enzyme inhibitory activity of these compounds (Table 5 and Figure 5).

**Table 2:** Weight of prostate gland of animals of different groups

**S. No.**	**Group No.**	**Treatment**	**Weight of prostate (gm)**
1.	I	Testosterone (s.c)+ vehicle (topical)	0.1094 ± 0.002
2.	II	Testosterone (s.c)+ 2% Finasteride solution (topical)	0.0992 ± 0.001*
3.	III	Testosterone (s.c)+ 2% Pet Ether Extract of *P. niruri* solution (topical)	0.1024 ± 0.003**

Values are mean ± SD, n=3

* p < 0.05,

** p < 0.001, significance versus control (Group I)

**Table 3:** Anti-hair loss concentrations of petroleum ether extract of *P. niruri*

**S. No.**	**Concentration of Extract (mg/ml)**	**Peak Area (sq. mm)**	**RTC** * ** (μg/ml)**	**Regressed Values**	**Statistical Analysis**
1.	0.85	519935	4.752	4.933	Correlation coefficient r^2^ =0.9534 Equation of Straight line y =1.9197x + 3.3022
2.	1.25	669444	5.973	5.701	
3.	1.75	734002	6.501	6.661	
4.	2.00	843932	7.398	7.141	
5.	2.25	848657	7.437	7.621	

* Relative Testosterone Concentration

**Table 4:** Inhibitory concentrations of finasteride

**S. No.**	**Concentration (mg/ml)**	**Peak Area (sq. mm)**	**RTC** * ** (μg/ml)**	**Regressed Values**	**Statistical Analysis**
1.	1	345803	3.330	3.674	Correlation coefficient r^2^ = 0.9174 Equation of Straight line y =1.3622x + 2.3125
2.	2	577971	5.226	5.036	
3.	3	801845	7.055	6.399	
4.	4	827107	7.261	7.761	

* Relative testosterone concentration

**Table 5:** IC50 value of extract and finasteride

**Treatment**	**IC50 values**
T + petroleum ether extract of *P. niruri*	1.46 mg/ml
T + finasteride	1.81 mg/ml

SD=0.2475

**Statistical analysis**

 All data presented as Mean± SD of at least three samples. Data were compared with control group by Student’s t-test using graph pad instat version 2 for windows. 

## Discussion

Discovery of novel hair growth promoters is of great importance as only two drugs namely minoxidil (topical) and finasteride (oral) have been approved by FDA for the treatment of alopecia (Kakali et al. 2009 ▶). Androgenetic alopecia is a dihydrotestosterone-mediated process, described by continuous miniaturization of androgen reactive hair follicles and accompanied by perifollicular fibrosis of follicular units in histological examination (Yoo et al. 2006 ▶). Androgenetic alopecia was induced in rats by administration of testosterone. Conversion of testosterone to dihydrotestosterone (DHT), which is a more potent androgen compared to testosterone causes miniaturization of hair follicle and shortening of the anagen phase and markedly prolongs the duration of resting phase or telogen resulting in conversion of thin terminal hairs into fine vellus hairs. The enzyme 5α-reductase type-2 is the key enzyme responsible for conversion of testosterone to dihydrotestosterone (Kaufman, 2002 ▶). In our work, this was the target to promote hair growth.

The enzyme 5α-reductase type-2 is mainly found in the prostate. The prostate homogenate revealed conversion of testosterone to dihydrotestosterone in reaction mixtures (Steers, 2001 ▶). *In vitro* study demonstrated 5-reductase inhibitory activity and also showed that conversion of testosterone to DHT was reduced. Increased testosterone level in reaction mixture was due to inhibition of 5-reductase because it is not converted to its metabolite dihydrotestosterone. Addition of petroleum ether extract of *P. niruri* and finasteride to reaction mixture increased the levels of unchanged testosterone in reaction mixture, suggesting inhibition of enzyme by these test materials probably due to the presence of lignans and terpenoids in extract. The rate of hair growth declines and the duration of the resting phase or telogen increases in rats due to androgens-induced alopecia. In normal animals, anagen telogen ratio is 6 to 8. In Androgenetic alopecia, the number of anagen follicle decreases whereas telogen follicles increase. That is the reason for the presence of more telogen follicles in group I. Testosterone-induced alopecia in rats was counteracted when extract was administered simultaneously. Alopecia was not observed in groups, which were treated with extract or finasteride along with testosterone. Anagen follicles were more than telogen follicles in petroleum ether and finasteride-treated groups. Due to 5α-reductase inhibitory activity of extract and finasteride, duration of anagenic follicles as well as follicular length increases, their miniaturization is prevented and transformation of a miniaturized, vellus-like hair back into a terminal one is stimulated (Whiting et al. 1999 ▶). In our study, hair growth promoting activity of the extract was confirmed by visual observation and quantitative data (e.g. A/T ratio and hair follicular density) in rats. 

It is concluded that petroleum ether extract of *P. niruri *acts as a hair growth-promoting agent in Androgenetic alopecia probably through inhibition of 5α-reductase enzyme and reduction of the conversion of testosterone to more potent compound, dihydrotestosterone in the skin. Dihydrotestosterone has been also shown to be responsible for other androgen-dependent conditions like benign prostatic hyperplasia, prostatic cancer, and acne (Pérez-Ordeals et al. 2005 ▶). The observed 5α-reductase inhibitory activity of the extract makes it a potential candidate in management of the above-mentioned conditions. 

## Conflict of interest

The authors declare that they have no conflict of interest.
